# Lingual juvenile xanthogranuloma in a woman: a case report

**DOI:** 10.1186/1752-1947-5-30

**Published:** 2011-01-24

**Authors:** Alessandro Villa, Umberto Mariani, Francesco Villa

**Affiliations:** 1 Department of Dentistry, Azienda Ospedaliera Ospedali Riuniti di Bergamo, Largo Barozzi 1. I-24128 Bergamo, Italy

## Abstract

**Introduction:**

Juvenile xanthogranuloma is a rare non-Langerhans cell histiocytosis that usually occurs during infancy and early childhood. The presence of single or multiple raised cutaneous lesions characterize this self-healing disorder. Extracutaneous sites are rare.

**Case presentation:**

We present a rare case of oral juvenile xanthogranuloma in a 49-year-old Caucasian woman. The histopathologic diagnosis of the lingual neoformation was histiocitary proliferation with the presence of giant cells, Touton type, compatible with juvenile xanthogranuloma.

**Conclusion:**

To establish an accurate diagnosis, microscopic evaluation and immunohistochemical staining are necessary. Dentists, dermatologists and general practitioners may be the first to recognize this rare condition during the inspection of the oral cavity.

## Introduction

Juvenile xanthogranuloma is an uncommon non-Langerhans cell histiocytosis that usually occurs during infancy and early childhood. This lesion was first reported by Adamson in 1905 [1], who used the term *congenital xanthoma multiplex*. This pathological condition has subsequently been reported under various names such as nevoxanthoendothelioma, juvenile xanthoma or xanthoma tuberosum [2]. Its modern name was introduced by Senear [3] in 1936 and popularized by Helwig and Hackney in 1952 [4].

The pathogenesis is unknown, although the disease is believed to be a reactive rather than a neoplastic process. It is caused by the proliferation of plasmacytoid monocytes in response to an unknown etiologic agent, possibly either physical or infectious [5].

Juvenile xanthogranuloma is a benign and self-healing disorder which is characterized by the presence of single or multiple raised cutaneous nodules, yellow-brown to reddish in color and usually measuring from a few millimeters to a few centimeters in diameter. Sometimes superficial telangiectasias and an erythematous border may also occur [6]. The most involved cutaneous regions are the neck, head and upper trunk, followed by the extremities.

Juvenile xanthogranuloma is not limited to cutaneous sites, however. The eye is the most common extracutaneous site, with other affected organs including the lung, kidney, pericardium, colon, central nervous system, liver, spleen, eye/orbit, bone, kidneys, adrenal glands, testis and ovary [7]. Treatment consists of surgical excision of the lesions, with or without radiation therapy or chemotherapy. The prognosis is usually good; however, systemic juvenile xanthogranuloma may be life-threatening [8]. According to Consolaro *et al. *[9], oral lesions are rare. Only 31 microscopically documented cases have been previously reported in the English-language literature. Moreover, among these 31 cases, only six occurred in patients above 18 years of age.

The aim of this report is to highlight an additional case of solitary extracutaneous juvenile xanthogranuloma involving the tongue in an adult patient.

## Case presentation

Approximately five years ago, a 49-year-old Caucasian woman came to our hospital for diagnosis, evaluation and treatment of a lingual lesion.

**Figure 1:**
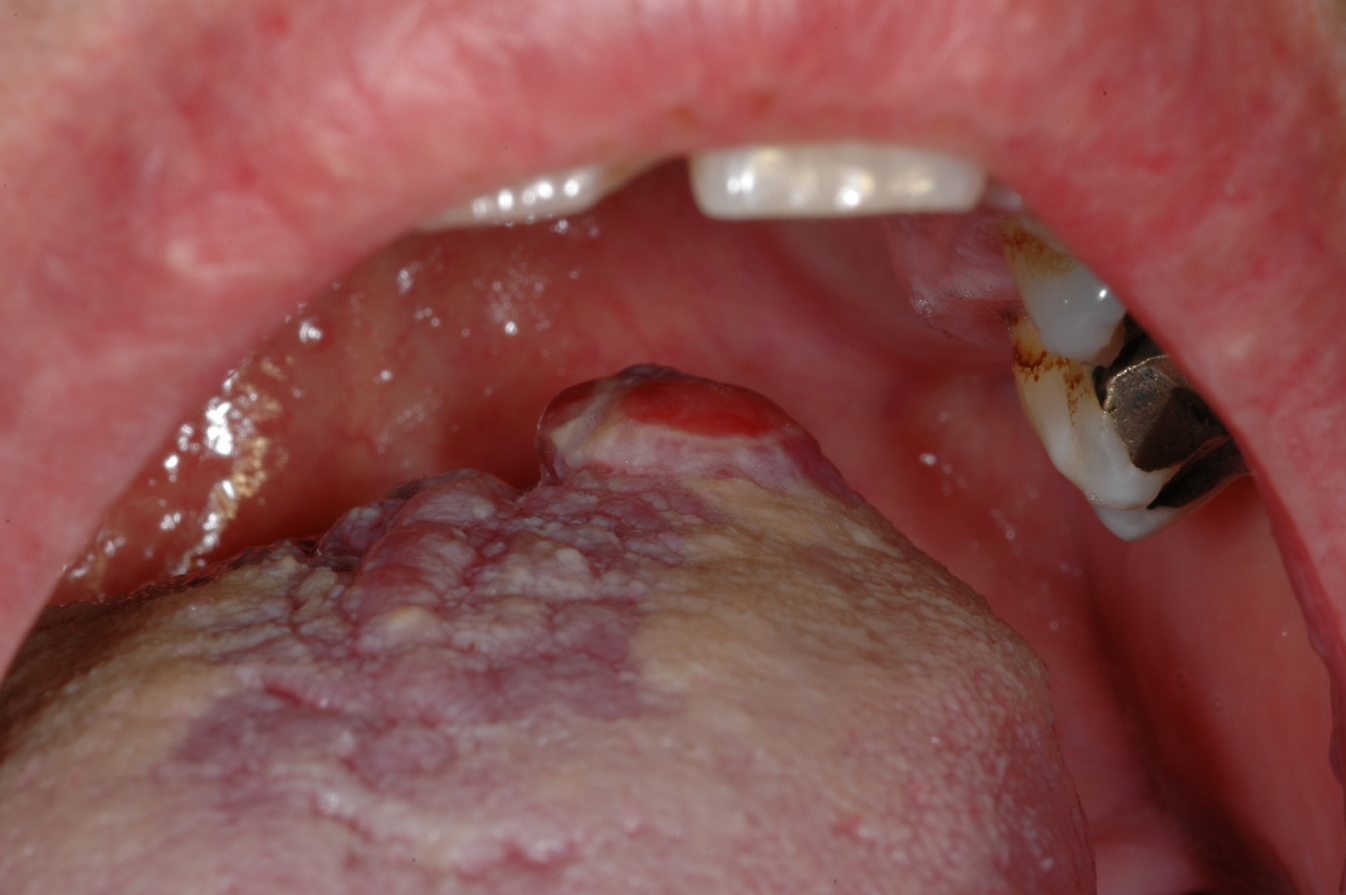
**Clinical presentation of the lesion**.

She complained of constant dysphagia and dysgeusia. These symptoms had worsened in the past three months because of growth of the lingual mass (Figure 1).

Her medical history was uneventful, except for breast cancer several years earlier, and included no history of smoking or drinking.

Clinical examination revealed the presence of a nodular neoformation with a fibrotic aspect on the dorsum of the tongue. Its consistency was hard, and it consisted of nodular, sessile microsurveys.

**Figure 2:**
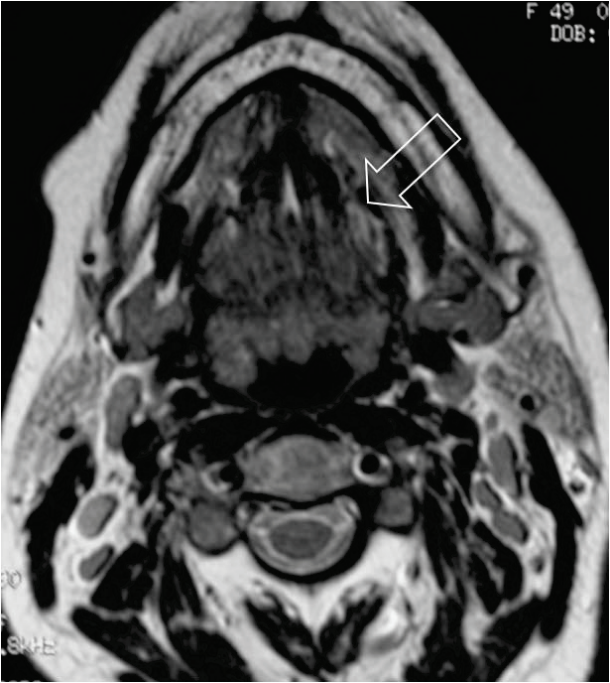
**Tongue magnetic resonance image showing the neoformation (arrow)**.

Because of the rapid clinical evolution of the lesion in a patient with previous breast cancer, the pathologist (UM) decided to perform an incisional biopsy immediately to exclude malignancy. Local anesthesia was administered deep around the proposed biopsy site. The specimen was taken with a scalpel to a depth of at least 5 mm, and a 3- to 5-mm margin of clinically normal mucosa was also included. The tissue was submitted in 10% neutral buffered formalin fixative for microscopic examination. A resorbable suture with polyglactin (Vicryl Rapide, Ethicon Ltd, Edinburgh, UK) was used. An axial magnetic resonance imaging (MRI) study was also requested for a diagnostic scope (Figure 2).

**Figure 3:**
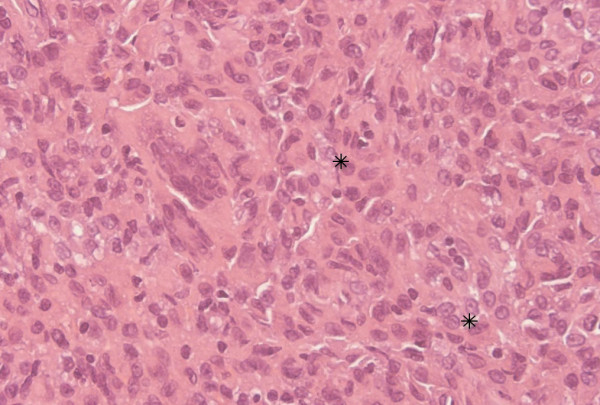
**Microscopic features show giant cells, Touton type (asterisk), and hematoxylin-eosin staining; original magnification ×400**.

The histopathologic diagnosis was histiocytic proliferation with the presence of giant cells, Touton type, compatible with juvenile xanthogranuloma (Figure 3).

After the diagnosis, the patient was included in a bimestrial follow-up program. During the first two months, we observed a modest volume reduction of the neoformation together with a reduction of its consistency.

**Figure 4:**
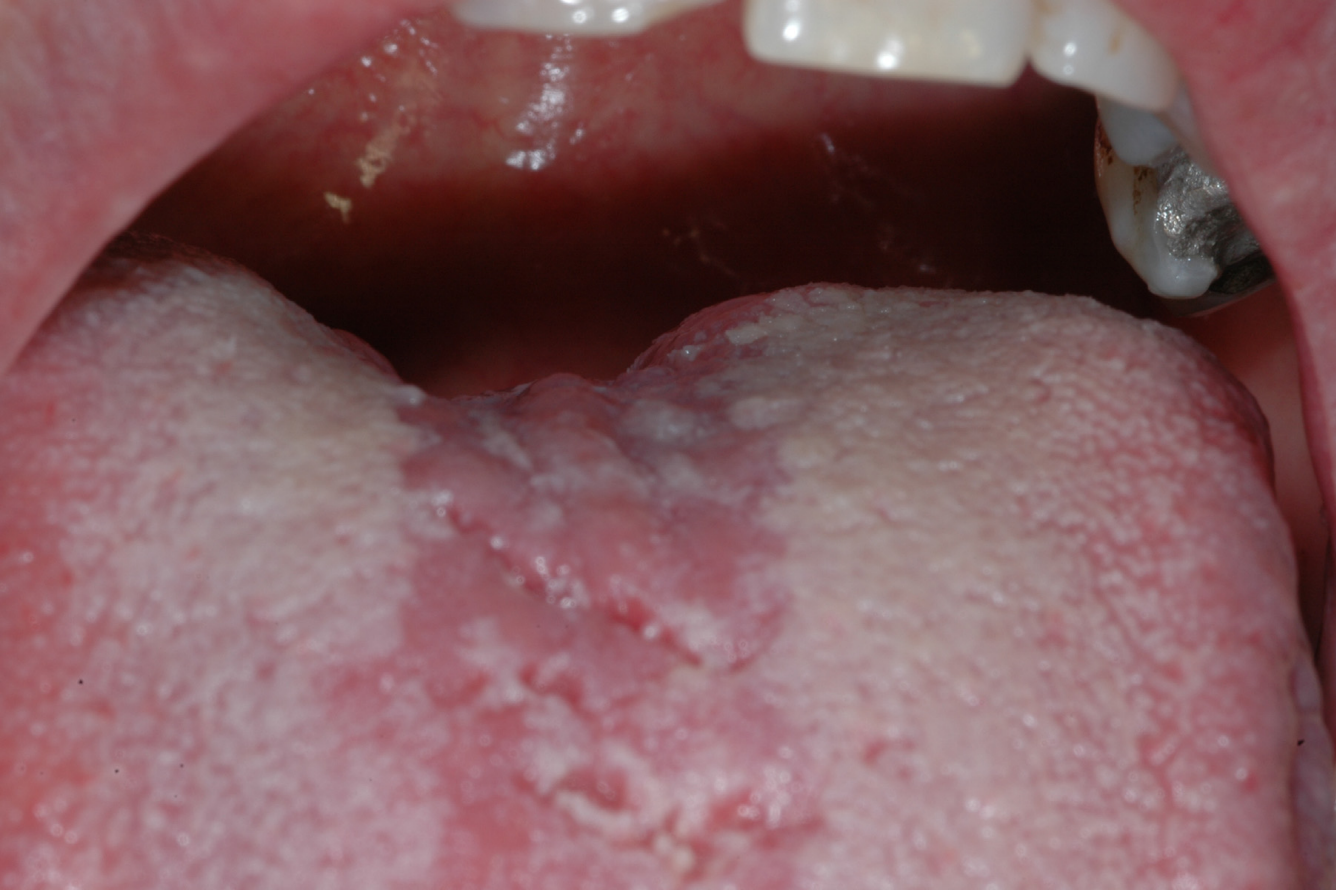
**Follow-up after more than four years**.

Afterward the lingual lesion underwent a spontaneous involution. To date, a hyperchromic area and a mild atrophy remain, without any evidence of local recurrence after follow-up of more than four years (Figure 4).

## Discussion

Juvenile xanthogranuloma is a benign disorder which characteristically develops as a well-demarcated mass in the dermis of a child. This disease heals spontaneously within one to several years in the majority of cases.

In most cases, cutaneous juvenile xanthogranuloma has a favorable prognosis and does not require any treatment at all, except for periodic follow-up until its disappearance [10]. Although spontaneous regression has not been reported in oral lesions [11], in the present case spontaneous healing was observed.

Of the 31 previously reported biopsy-proven cases of juvenile xanthogranuloma with onset in the oral cavity, only eight occurred on the tongue. After the biopsy, all patients did well. Recurrence after partial or complete excision of lesions has been reported in previous studies [12].

Because juvenile xanthogranuloma in the oral cavity is extremely rare and usually unifocal, it may be difficult to diagnose. Because its clinical appearance may be quite similar to dental abscess giant cell fibroma, gingival cyst, fibroma, peripheral ossifying fibroma, peripheral giant cell granuloma, peripheral odontogenic fibroma, pyogenic granuloma and lipoma, the only way to establish an accurate diagnosis is through microscopic examination, including immunohistochemical staining. A close follow-up after initial biopsy may be preferable to aggressive resection of the lesion when histological diagnosis is unequivocal.

General practitioners, dermatologists and dentists may be the first health care professionals to see patients with symptoms and signs of oral disease, which should be referred to an oral pathologist and oral surgeon for diagnosis and treatment.

## Conclusions

We have presented a rare case of lingual juvenile xanthogranuloma. In our opinion, although gross total resection of lingual xanthogranuloma is regarded as curative, one may decide alternatively to follow up closely for spontaneous regression after initial biopsy.

## Competing interests

The authors declare that they have no competing interests.

## Consent

Written informed consent was obtained from the patient for publication of this case report and accompanying images. A copy of the written consent is available for review by the Editor-in-Chief of this journal.

## Authors' contributions

AV analyzed and interpreted the patient data regarding the oral lesion and the histological examination. UM performed the biopsy. FV reviewed the manuscript and followed the patient together with AV and UM during the more than four years of follow-up. All authors read and approved the final manuscript.
